# Implementation of absolute quantification in small‐animal SPECT imaging: Phantom and animal studies

**DOI:** 10.1002/acm2.12094

**Published:** 2017-05-16

**Authors:** Shabnam Khorasani Gerdekoohi, Naser Vosoughi, Kaveh Tanha, Majid Assadi, Pardis Ghafarian, Arman Rahmim, Mohammad Reza Ay

**Affiliations:** ^1^ Department of Energy Engineering Sharif University of Technology Tehran Iran; ^2^ Research Center for Molecular and Cellular Imaging Tehran University of Medical Sciences Tehran Iran; ^3^ The Persian Gulf Nuclear Medicine Research Center Bushehr University of Medical Sciences Bushehr Iran; ^4^ Chronic Respiratory Diseases Research Center National Research Institute of Tuberculosis and Lung Diseases (NRITLD) Shahid Beheshti University of Medical Sciences Tehran Iran; ^5^ PET/CT and Cyclotron Center Masih Daneshvari Hospital Shahid Beheshti University of Medical Sciences Tehran Iran; ^6^ Department of Radiology Johns Hopkins University Baltimore Maryland USA; ^7^ Department of Electrical and Computer Engineering Johns Hopkins University Baltimore Maryland USA; ^8^ Departmen of Medical Physics and Biomedical Engineering Tehran University of Medical Sciences Tehran Iran

**Keywords:** attenuation correction, quantification, small‐animal imaging, SPECT imaging

## Abstract

**Purpose:**

Presence of photon attenuation severely challenges quantitative accuracy in single‐photon emission computed tomography (SPECT) imaging. Subsequently, various attenuation correction methods have been developed to compensate for this degradation. The present study aims to implement an attenuation correction method and then to evaluate quantification accuracy of attenuation correction in small‐animal SPECT imaging.

**Methods:**

Images were reconstructed using an iterative reconstruction method based on the maximum‐likelihood expectation maximization (MLEM) algorithm including resolution recovery. This was implemented in our designed dedicated small‐animal SPECT (HiReSPECT) system. For accurate quantification, the voxel values were converted to activity concentration via a calculated calibration factor. An attenuation correction algorithm was developed based on the first‐order Chang's method. Both phantom study and experimental measurements with four rats were used in order to validate the proposed method.

**Results:**

The phantom experiments showed that the error of −15.5% in the estimation of activity concentration in a uniform region was reduced to +5.1% when attenuation correction was applied. For *in vivo* studies, the average quantitative error of −22.8 ± 6.3% (ranging from −31.2% to −14.8%) in the uncorrected images was reduced to +3.5 ± 6.7% (ranging from −6.7 to +9.8%) after applying attenuation correction.

**Conclusion:**

The results indicate that the proposed attenuation correction algorithm based on the first‐order Chang's method, as implemented in our dedicated small‐animal SPECT system, significantly improves accuracy of the quantitative analysis as well as the absolute quantification.

## 1 INTRODUCTION

Preclinical single‐photon emission computed tomography (SPECT) imaging is significantly utilized in present day imaging and research.^1^, ^2^, ^3^ Quantification can expand capabilities of SPECT and play a significant role in drug development,^4^, ^5^ organ dosimetry,^6^ and therapy response assessment.^7^ The term “quantification” can actually have one of several meanings including (a) semiquantification where relative measurements are compared with disease‐free regions, (b) physiological quantification such as perfusion or glucose metabolic rate, and (c) absolute quantification which is the main focus in this paper: the measurement of the true activity concentration in a volume of interest.^8^

Several small‐animal SPECT systems have been developed in recent years,^9^, ^10^, ^11^, ^12^, ^13^, ^14^, ^15^ and there are some publications involving absolute quantification in preclinical SPECT imaging. The finding of Vanhove et al.^16^ showed that quantitative errors in mice experiments could be reduced to −7.9 ± 10.4% when attenuation and scatter corrections were applied. In another work, Wou et al.^17^ developed a method for attenuation correction to improve the absolute quantification for the U‐SPECT‐II, a stationary multipinhole SPECT system for small‐animal imaging. In their phantom experiments, a quantification error of −18.7% was reduced to −1.7% when including both scatter and attenuation corrections; by contrast, in animal experiments, the errors were between −6.3% and +4.3%. In 2011, Finan et al.^18^ investigated the quantitative accuracy of the NanoSPECT system using phantom and animal models. The absolute quantification error for Tc‐99m and In‐111 *in vivo* studies were within 12% of the true activity. Another study by Vandeghinste et al.^19^ demonstrated the feasibility of *in vivo* absolute quantification of the trimodal FLEX Triumph‐II system, while a quantification error less than 5% was achieved. In their study, image reconstruction included scatter correction and CT‐based attenuation correction within image reconstruction. CT‐based attenuation correction provided a more accurate attenuation map, but at the cost of needing additional hardware.^4^ However, due to the small size of mice, a uniform attenuation map may be sufficient for quantification.

According to some studies, there are physical degradation factors which have been compensated for, such as photon attenuation^20^, ^21^ and scatter.^22^, ^23^ Photon attenuation correction is imperative for improving the quantitative accuracy.^24^, ^25^ For instance, attenuation can reduce the measured activity concentration in a volume of interest in the center of a rat‐sized cylinder water phantom by up to 25% when imaging with Tc‐99m.^24^ Scattering in body can also decrease the quantitative accuracy, but this error is negligible in objects with the size of small animals as imaged using Tc‐99m.^24^

The aim of this study is to develop and evaluate an approach to quantitative SPECT imaging using an iterative reconstruction method along with corrections. Specifically, we aim to improve the quantification accuracy in our dedicated animal SPECT system called HiReSPECT.^26^, ^27^, ^28^ We have developed a calibration method to determine the activity concentration in MBq/ml, via a system image matrix. We also used a rotation‐based MLEM reconstruction algorithm with resolution recovery and postreconstruction attenuation correction algorithms. Various phantom studies were designed to evaluate the accuracy of quantification, and finally some animal experiments were undertaken to validate our method for *in vivo* imaging.

## 2 MATERIALS AND METHODS

### 2.A The HiReSPECT scanner

**Figure 1:**
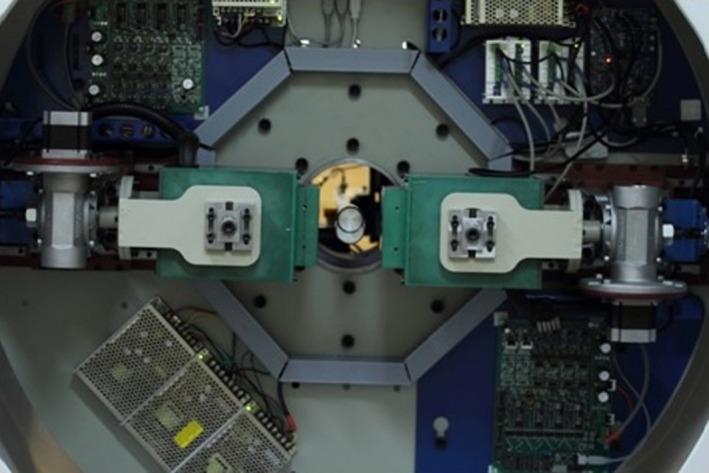
The gantry of the HiReSPECT system developed in our department includes two heads, an animal bed and a data acquisition board.

The HiReSPECT scanner^28^ is a newly developed system in our department, Tehran University of Medical Science, which is designed for small‐animal SPECT imaging (Fig. 1). It is a dual‐head system, and each head has a pixelated CsI(Na) crystal (Hilger Crystals, UK) consisting of a 46 × 89 array of pixels. The size of each pixel is 1 × 1 × 5 mm^3^, and there is a 0.2‐mm epoxy septum between the pixels, thus having a pixel pitch of (1.2 mm)^2^, and the active area of the crystals is about 10 × 5 cm^2^. Given the active area, each projection is saved as a 38 × 80 matrix. Each head has a high‐resolution parallel‐hole collimator (Nuclear Fields Co., Australia). The face‐to‐face distance, septa size, and thickness of the holes are 1.2, 0.2, and 34 mm, respectively. A pair of H8500C PMTs (Hamamatsu Photonic Co., Japan) is fixed on the scintillation crystal. The system gantry can rotate around a carbon fiber bed, and its radius of rotation can be changed. Decay compensation is also included during system data acquisition.

### 2.B Image reconstruction and correction

#### 2.B.1 Reconstruction algorithm

Image reconstruction of the system is based on the maximum‐likelihood expectation maximization (MLEM) algorithm, and the final reconstructed image is a 3D 128 × 128 × 240 matrix. The system reconstruction software uses a rotation‐based algorithm to speed up the reconstruction process.^27^ The collimator‐detector response function (CDRF) of the system was modeled, and a resolution recovery algorithm was embedded within the reconstruction code.^27^ CDRFs were measured based on a pixel‐driven method using six capillary sources. The CDRFs were fitted on independent 2D Gaussian functions at various distances from the head.

#### 2.B.2 Attenuation correction

Since attenuation correction is essential in quantitative SPECT, a practical first‐order attenuation correction algorithm was used to compensate for this degrading physical effect. There exist a number of attenuation correction methods, which can be categorized into preprocessing, iterative, and postprocessing techniques.^29^ For the present work, a postprocessing technique was used so that a new image matrix is not required for each object. A first‐order Chang's attenuation correction algorithm^30^ that assigns a uniform linear attenuation coefficient to all of the object points was used. The first step was to determine the contour of the object. Since this version of the HiReSPECT does not have a CT scanner, the determination of the object contour was performed using emission data.^31^ To determine the edges of the object in the image, an algorithm was developed based on thresholding. In this method, the fraction of transmitted photons along each view is calculated for a given voxel *i*, and the values are averaged for all views, as follows:

(1) $$TF_{i}=\frac{1}{M}\sum_{m=1}^{M}exp\left(-\mu .l_{m,i}\right)$$

where *M* is the number of views, *μ* is the attenuation coefficient set to 0.15 cm^−1^ (attenuation coefficient of water at 140 keV; energy of ^99m^Tc photons), and $l_{m,i}$ is the distance between the given voxel *i* and the object edge along a line perpendicular to the surface of the detector at view *m*. The value of *M* was set to 60, that is, the number of system data acquisition views. The *TF* coefficients were calculated for all voxels in the image matrix.

#### 2.B.3 System calibration

In the SPECT image matrix, voxel values show the number of counts that were received to the detector and do not provide quantitative information about the amount of radiopharmaceutical present in an object. An experiment was undertaken to achieve a calibration factor (CF) for converting the voxel values to an activity concentration with the dimensions MBq/ml. A point source with known activity was scanned and reconstructed, and the system calibration factor was given by the following formulation after decay correction:

(2) $$CF=\frac{A}{V.\sum^{R}}$$

where *A* is the point source activity measured in the dose calibrator, *V* is the volume of a voxel, and *ΣR* is the summation of all voxel values in the reconstructed image. To compute the activity concentration (AC), including attenuation compensation for any given voxel *i* of value $R_{i}$, the following formula was used:

(3) $$AC_{i}=\frac{R_{i}.CF}{TF_{i}}$$

As it can be seen, the attenuation compensation coefficient and the calibration factor act as scaling factors. In fact, they eliminate the need to define a new system matrix and a new reconstruction algorithm given the postprocess nature of the operation.

#### 2.B.4 Sensitivity correction

Five point sources were scanned with scan parameters listed in Table 1, where their activities were measured with a calibrated dose calibrator (42.7 ± 0.1 MBq) using a calibrated source by a national standards laboratory. Point sources in air were chosen because they are nearly attenuation and scatter free; therefore, one does not need to add these compensations to the reconstruction. All data acquisition and reconstruction parameters were matched for these scans except for the radius of rotation (ROR) which is considered as half the distance between the system detector surfaces. Since the detection sensitivity in parallel‐hole collimators is not independent of the distance between the source and detector surface, a small decrease is observed when the ROR is increased and thus the calibration factor was calculated for various RORs.

**Table 1:** SPECT data acquisition parameters for phantom and animal scans

Parameter	Setting	
	Phantom studies	Animal studies
Data acquisition	Dual head	Dual head
Radius of rotation	30,40, 50, 60, and 70 mm	50 mm
Mode	Step and shoot	Step and shoot
Number of projections	120	120
Angular step	6°	6°
Time per view	30–300 s	30 s
Projection matrix	38 × 80	38 × 80
Image matrix	128 × 128 × 240	128 × 128 × 240
Voxel size	0.4 × 0.4 × 0.4 mm^3^	0.4 × 0.4 × 0.4 mm^3^

### 2.C Phantom experiments

#### 2.C.1 Point sources

Sixteen point sources were made and scanned with the same data acquisition parameters, and their images were reconstructed. Prior to scanning, the activities were measured with a dose calibrator (in a range of 18.6–283.3 MBq) and were also obtained from the calibrated image matrix. The correlation between these two ways of quantification (dose calibrator vs. imaging) was examined.

#### 2.C.2 Cylindrical tubes

In nuclear imaging, the partial volume effect reduces the accuracy of quantification.^32^, ^33^, ^34^, ^35^, ^36^ This means that as the target diminishes in size, the estimated activity will be less than the true value. To evaluate this phenomena in the present system, a cylindrical tube with six different volumes (1, 2.5, 5, 10, 20, and 30 ml) was scanned with the same data acquisition parameters and their estimated activities from the images were compared with the known values. These cylindrical tubes had outer diameters of 7, 11, 15, 20, 26, and 32 mm. The images were reconstructed with and without attenuation correction, and the results were subsequently compared.

#### 2.C.3 Image quality phantom

**Figure 2:**
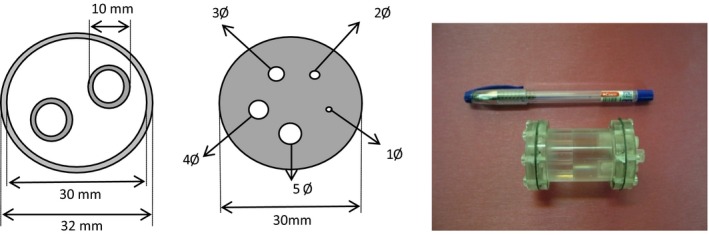
The image quality phantom recommended by NEMA NU‐4 2008.

To evaluate the quantitative accuracy in more complex objects, the national electrical manufacturers association (NEMA) NU 4‐2008 image quality phantom for small‐animal imaging (Fig. 2) was scanned. This phantom includes three parts: five holes (hot rods) with diameters of 1, 2, 3, 4, and 5 mm, a uniform region, and two cavities with an inner diameter of 8 mm and a length of 15 mm (cold region), which are filled with nonradioactive water and air. The main body of this phantom has an inner diameter of 30 mm. The phantom was filled with Tc‐99m solution with a concentration of 13.9 MBq ml^−1^ and scanned with ROR of 50 mm and a time per view equal to 30 s. In order to evaluate the system quantification as well as the impact of postprocessing attenuation correction algorithm on the quantitative accuracy, vertical and horizontal line profiles of the centered transverse slice of the uniform region were drawn. Furthermore, the quantitative accuracy of the system in an activity determination of focal hot spots was examined. Activity concentrations of the hot rods were obtained from the reconstructed images before and after attenuation correction, and were then compared with the measured activity.

### 2.D Animal experiments

Four female rats (eight kidneys) were used to test the quantitative accuracy, and attenuation correction algorithm as applied to a nonuniform distribution of attenuation coefficients in the body. Tc‐99m‐DMSA (dimercaptosuccinic acid) was used for renal imaging.^37^ For animal models, anesthesia was performed using a combination of xylazine 2% and ketamine 10% through a subcutaneous injection. After anesthesia, an injection of radiopharmaceutical (111 MBq for each animal) was delivered via the tail vain. Two hours later, the scans were completed with the data acquisition parameters listed in Table 1. To assess the real accumulated activity, the animals were autopsied following euthanasia via an overdose of anesthetics^38^ and the kidneys were removed to be counted with the dose calibrator. Decay compensation was applied in order to get the activities at the start of data acquisition. The values of the activity obtained from the measurement and reconstructed images were then compared.

### 2.E Data analysis

In order to perform quantitative analysis, volumes of interest (VOIs) were drawn and assessed. In phantom studies, VOIs were drawn manually with a cylindrical shape because there was no background activity; edges were sharp and the shapes were clear and simple to define. However, in animal studies, these conditions do not exist, and thus, a 3D isocontour method was used. The minimum count defining the VOI via thresholding was given by the following equation^39^:

(4) $$C_{min}=\left(C_{max}-C_{b.g.}\right)\times f+C_{b.g.}$$

where *C*_max_ is the maximum count in the area of interest, *C*_b.g._ is mean count surrounding the tissue, and *f* is a fractional value. Various amounts for *f* were experimented, and the best results were achieved when *f* was equal to 0.2 (20%). As such, all results were obtained by drawing VOIs with this factor. The analyses were performed with the open source AMIDE software package (version 1.0.4).

## 3 RESULTS

### 3.A Phantom study

**Figure 3:**
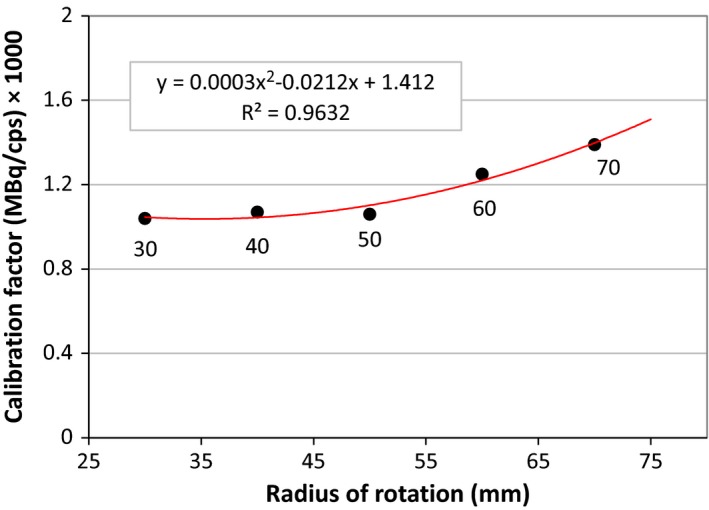
Dependence of system calibration factor on the radius of rotation of the detectors.

Calibration factors (CF) were obtained for five RORs and a function was fitted on these data; as a result, we obtained CF for any other RORs that will be used in data acquisition. Figure 3 shows the CF for converting voxel values to the activity concentration (MBq/ml) in various RORs.

**Figure 4:**
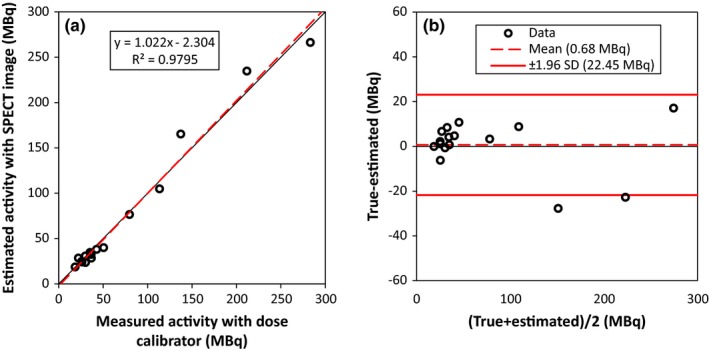
(a) Comparing point source activities measured with the dose calibrator and estimated from reconstructed images, (b) Bland–Altman plot of these data.

The relationship between measuring activities with a dose calibrator and using the reconstructed image of the point sources is shown in Fig. 4(a) (*R* ^2^ = 0.96). To obtain the activity from the images, we converted the voxel values to an activity concentration at first and then quantified the activity of the point sources using a large ROI drawn around their images. The difference between the measured activity of sources by using the dose calibrator versus imaging was statistically not significant (*P* = 0.81, a two‐tailed *T*‐test). The quantification relative errors were obtained to be −22.9% and +27.7% with an average of −3.7 ± 13.6%. Bland–Altman plot was also drawn as depicted in Fig. 4(b) to investigate the agreement between our gold standard (measured activity using the dose calibrator) and measurement by the image matrix. An underestimation of 0.68 MBq was observed.

**Figure 5:**
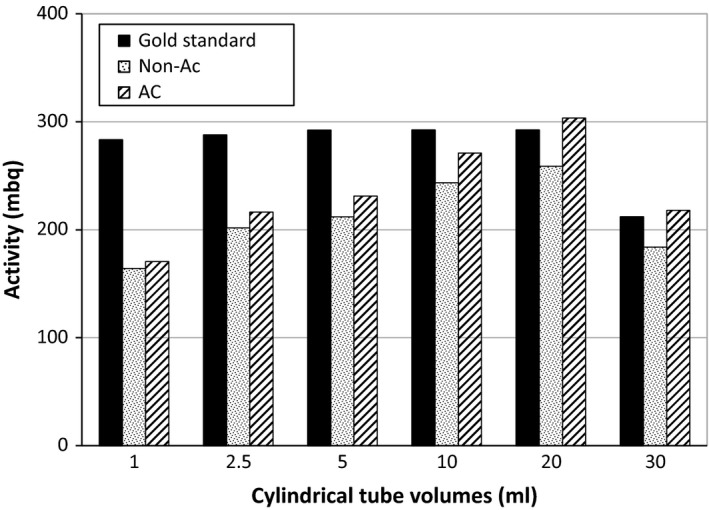
Measured and estimated activities of the six cylindrical tubes with different volumes that were scanned with the same data acquisition parameters.

Figure 5 summarizes the measured and estimated activities, and percentage relative errors between them, for cylindrical tubes with various volumes. It was found that the quantification relative error decreases from −42.0% to −13.1% by increasing the volume of the object from 1 to 30 ml. Also, the effect of attenuation correction on the improvement of relative error increased from 2.2% to 15.9% when the diameter of target increases from 7 to 32 mm. Besides, the difference between the measured activity of tubes by using a dose calibrator and calculating it using the images after attenuation correction was statistically not significant (*P* = 0.10, a two‐tailed *T*‐test). As it can be seen, the effect of attenuation correction on objects with various sizes are different; in fact, the amount of photon attenuation increases in larger objects, thus attenuation compensation becomes more important and more effective in enhancing the quantitative accuracy in these objects.

**Figure 6:**
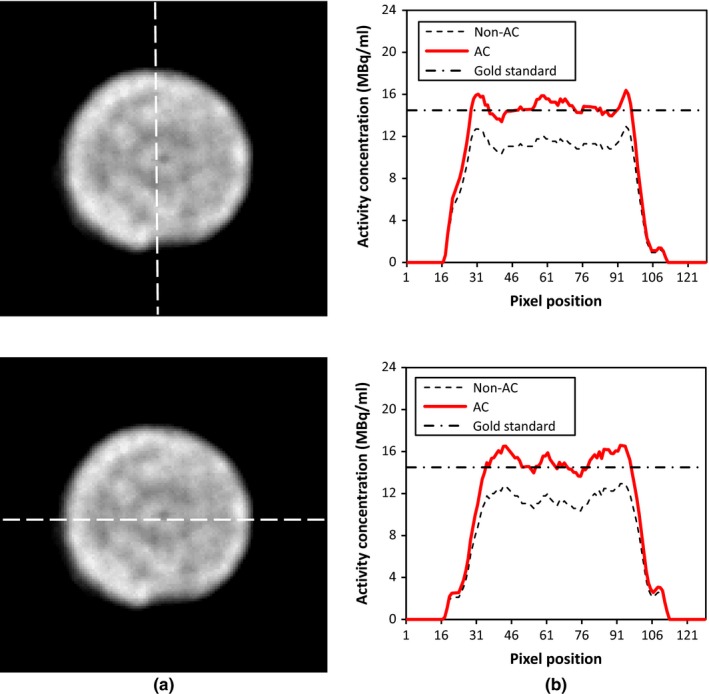
(a) Transverse slice of uniform region and (b) vertical and horizontal line profiles. AC: attenuation corrected, Gold standard: measured with the dose calibrator.

Figure 6(b) shows line profiles through the transverse slice in the uniform region of the phantom, before and after applying attenuation correction. The quantification error in the determination of activity concentration of this slice decreased from −15.5% to +5.1% with attenuation compensation. As can be seen, the underestimation in the activity concentration has been compensated for after attenuation correction. However, a slight activity overestimation is an anticipated consequence of the first‐order Chang's attenuation correction method.^17^

**Figure 7:**
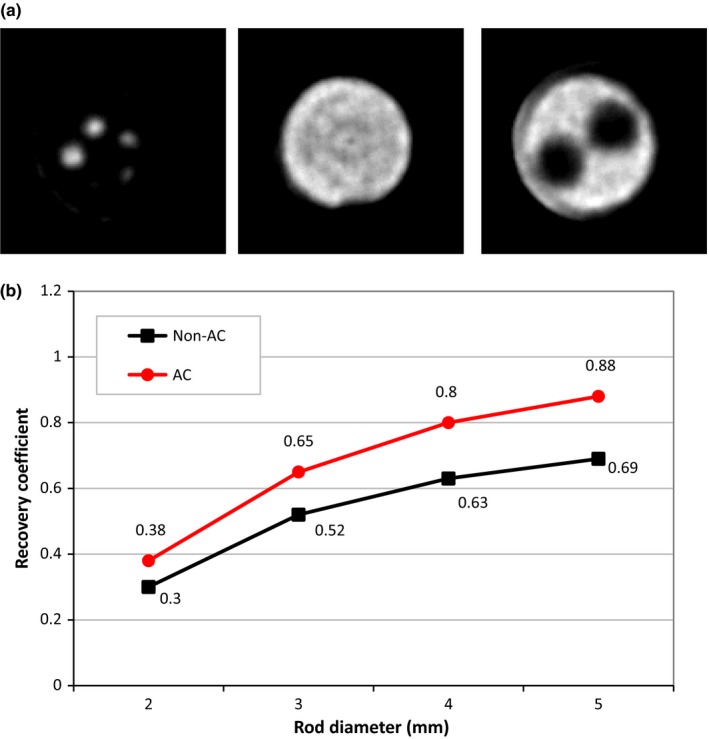
(a) Transverse slices of a reconstructed image of the image quality phantom and (b) recovery coefficient of the hot rods before and after applying attenuation correction.

Figure 7 depicts the recovery coefficients of the hot rods with diameters of 2, 3, 4, and 5 mm. The rod with the diameter of 1 mm is not detectable due to its size and is smaller than the spatial resolution of the system which has been reported equal to 1.7 mm at the ROR of 25 mm.^26^ Recovery coefficients of the activity concentration were achieved in both corrected and uncorrected images. The influence of attenuation correction on the recovery coefficient is noticeable with a maximum difference of 19% between the uncorrected and corrected images.

### 3.B Animal study

**Figure 8:**
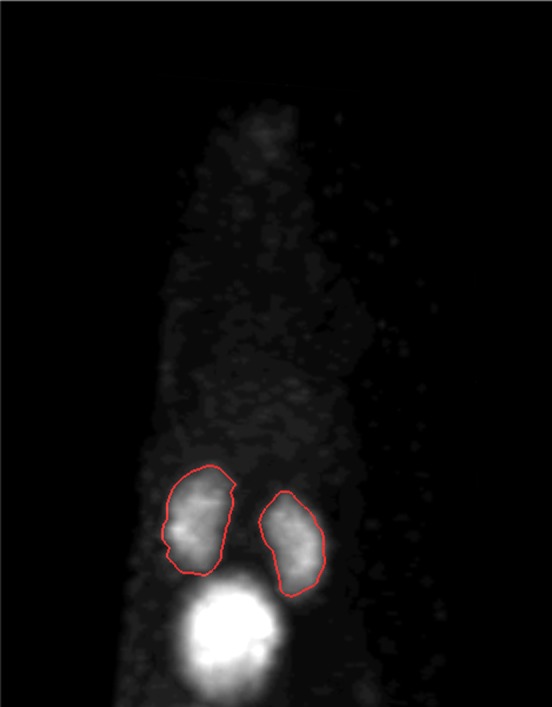
Representative animal images showing isocontour VOI drawn around the kidneys for quantitative analysis.

**Figure 9:**
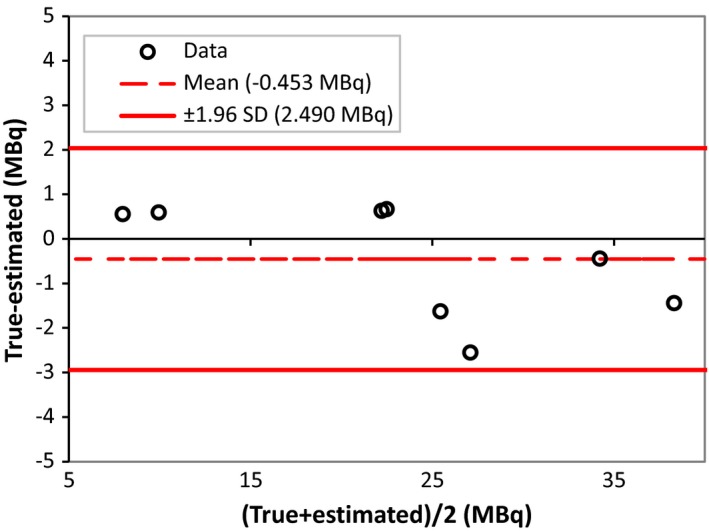
Bland–Altman plot comparing estimated kidney activity determined by SPECT images to activity measured in the dose calibrator.

A rat's kidney image with drawn VOIs is shown in Fig. 8. The isocontour around each kidney has been drawn using eq. 4 for calculating the absolute activity from images. Table 2 lists the activities of the kidneys measured in the dose calibrator and their quantitative results calculated on the uncorrected and attenuation corrected images. Figure 9 shows a Bland–Altman plot which is performed to illustrate the agreement between the gold standard and the activity measured by the SPECT images. The quantification errors on the uncorrected images (Non‐AC data in Table 2) ranged from −31.2% to −14.8% with an average of −22.8 ± 6.3%. With attenuation correction (AC data in Table 2), these errors ranged from −6.7% to +9.8% with an average of +3.5 ± 6.7% over all the kidneys investigated. The difference between relative errors in uncorrected and attenuation‐corrected images is statistically significant (*P* < 0.001); therefore, performing attenuation correction can bring about a very considerable and significant improvement to the quantitative accuracy.

**Table 2:** Activity of kidneys. Gold standard: measured with dose calibrator, Non‐AC: no attenuation correction was performed, AC: attenuation correction was performed

Kidney	Gold standard (MM)	Non‐AC		AC	
		Estimated activity	Relative error %	Estimated activity	Relative error %
Rat 1, left	25.826	21.978	−14.8	28.379	+9.8
Rat 1, right	24.642	20.831	−15.4	26.27	+6.6
Rat 2, left	8.251	6.290	−23.7	7.696	−6.7
Rat 2, right	10.249	7.363	−28.1	9.657	−5.8
Rat 3, left	22.829	18.278	−19.9	22.163	+9.7
Rat 3, right	22.533	18.019	−20	21.904	+9.7
Rat 4, left	37.592	25.826	−31.2	39.035	+3.8
Rat 4, right	34.003	23.865	−29.8	34.447	+1.3

## 4 DISCUSSION

Quantitative small‐animal SPECT imaging has an important role in radiolabeled drug development research because measuring the exact amount of concentrated activity in organs is mandatory. Compensation for degrading factors such as photon attenuation within the body is essential for achieving accurate quantification. In this study, we converted the image matrix voxel values from the count to activity concentration (MBq/ml), and then performed various phantom and animal studies to evaluate the quantitative accuracy of the proposed method. Specifically, a first‐order Chang's attenuation algorithm was used and its effect on the quantitative errors was evaluated.

In point source scans, no statistically significant differences were observed between the activities measured on the reconstructed images and those measured with the dose calibrator in the absence of attenuation. The same dose calibrator was used for system calibration and to measure the activity of the sources, with the aim of eliminating the bias of inaccuracy of the dose calibrator.

As Fig. 5 shows, when an attenuation correction is performed, the quantitative errors are reduced. Moreover, the attenuation compensation becomes more important as objects increase in size and self‐attenuation increases.

The NEMA image quality phantom study showed that the accuracy of quantification is acceptable when more complex objects with nonuniform activity concentrations are used. Attenuation correction could reduce the relative error of the activity concentration in the uniform region (Fig. 5) from −15.5% to +5.1%, whereas Wu et al.^40^ reduced this error from −16.3% to +4.8% with a CT‐based nonuniform attenuation correction algorithm. Thus, our finding is consistent with the finding of Wu et al. In hot rods of the image quality phantom, we achieved a recovery coefficient of 88% for the rod with a diameter of 5 mm, which is better than the Magota et al.^41^ In their study, the performance evaluation of the Inveon PET/SPECT/CT system reported a recovery coefficient of ~85% for the SPECT modality. By contrast, the authors detected the 1 mm hot rod because of using a pinhole collimator instead of our parallel‐hole collimation.

Quantitative analyses on renal scan images of animal models were undertaken using our optimized method for drawing VOIs around the kidneys making use of emission data only. The experimental data showed that photon attenuation correction reduces the quantitative error by 26.4% on average. Accordingly, and as already mentioned, attenuation correction is a vital and significant factor for achieving accurate quantification in small‐animal SPECT imaging.

In small objects (small animals), attenuation correction causes a little overestimation in the results, which is a normal consequence of using first‐order Chang's correction, and if one compensates for the effect of photon scattering, this overestimation will be removed.^17^ Therefore, in future work, we can implement scatter correction to achieve even more accurate quantification.

In the present study, we performed an experimental preclinical study only involving a renal scan; however, for future studies, we plan to investigate the quantitative accuracy on tumors and other organs with Tc‐99m and other radionuclides.

An important advantage of the present framework is that a new system matrix is not required in order to add the quantitative analysis capability to the system. As such, we significantly improve the quantitative accuracy of our system simply with a postprocessing attenuation correction method, instead of iterative methods. Moreover, our attenuation correction method not only provides acceptable quantitative accuracy, but also is very practical and independent from an additional hardware to determine the attenuation map.

## 5 CONCLUSION

We have implemented a method for attenuation correction of emission data, as applied to HiReSPECT, a dedicated small‐animal imaging system. This method improves the quantitative accuracy of images provided by this system. We also validated the proposed method using an experimental study in both phantom and animal models. Attenuation compensation was able to effectively and significantly reduce the relative error in the estimation of activity concentration in the uniform region of the NEMA image quality phantom from −15.5% to +5.1%. The *in vivo* kidney studies yielded a relative error of +3.5 ± 6.7% in the absolute quantification of activity. The results showed that the investigated method can be used for routine attenuation correction in SPECT systems in order to improve the accuracy of quantitative analysis.

## ACKNOWLEDGMENTS

This work was supported by the Research Center for Molecular and Cellular Imaging (RCMCI), Tehran University of Medical Science under Grant No. 24166 and Masih Daneshvari Hospital, Shahid Beheshti University of Medical Sciences, Tehran, Iran.
